# Dual-Band Operation of a Microstrip Patch Antenna on a Duroid 5870 Substrate for Ku- and K-Bands

**DOI:** 10.1155/2013/378420

**Published:** 2013-12-05

**Authors:** M. M. Islam, M. T. Islam, M. R. I. Faruque

**Affiliations:** Institute of Space Science (ANGKASA), Universiti Kebangsaan Malaysia UKM, 43600 Bangi, Selangor D. E., Malaysia

## Abstract

The dual-band operation of a microstrip patch antenna on a Duroid 5870 substrate for Ku- and K-bands is presented. The fabrication of the proposed antenna is performed with slots and a Duroid 5870 dielectric substrate and is excited by a 50 Ω microstrip transmission line. A high-frequency structural simulator (HFSS) is used which is based on the finite element method (FEM) in this research. The measured impedance bandwidth (2 : 1 VSWR) achieved is 1.07 GHz (15.93 GHz–14.86 GHz) on the lower band and 0.94 GHz (20.67–19.73 GHz) on the upper band. A stable omnidirectional radiation pattern is observed in the operating frequency band. The proposed prototype antenna behavior is discussed in terms of the comparisons of the measured and simulated results.

## 1. Introduction

The implementation of the microstrip patch antenna is a milestone in wireless communication systems and is continuing to fulfill the changing demands of the new generation of antenna technology. Microstrip patch antennas are widely used in wireless communication systems because they are low profile, of light weight, of low cost, of conformal design, and easy to fabricate and integrate. Many researchers have heavy interest in designing Ku- and K-band antennas and still face a major challenge to implement these applications. The patch is the dominant figure of a microstrip antenna; the other components are the substrate and ground, which are the two sides of the patch [1]. Many dual-band antennas have been improved to face the rising demands of a modern portable wireless communication device that is capable of integrating more than one communication standard into a single system. For this reason, different types of antenna designs have been proposed [2–14].

A dual polarized microstrip patch antenna has been proposed for Ku-band applications with dimensions of 15 mm × 15 mm, and such an antenna has achieved a 950 MHz bandwidth with a maximum gain of 7.6 dB, as noted in [15]. In [16], a multiband patch antenna was designed for Ku- and K-band applications with dimensions of 8 mm × 10 mm, a bandwidth of 760 MHz, and a peak gain of 4.5 dB. In [17], a Ku-band patch antenna using notches and slit was proposed, whose dimensions are 7.6 mm × 10 mm, with a substrate thickness of 0.8 mm; Teflon is used as the dielectric substrate material, and the antenna obtained a maximum bandwidth of 600 MHz. In [18], a dual-band compact microstrip antenna was proposed for Ku-band applications using three pairs of thin slits from the sides of a rectangular patch, whose dimensions are 9.5 mm × 10 mm; Rogers RT/Duroid 5880 is used as the dielectric substrate material, with a substrate thickness of 0.254 mm; the antenna obtained a maximum bandwidth of 90 MHz. In [19], a dual-frequency triangular slotted microstrip patch antenna was proposed for Ku-band applications, where the patch dimensions are 8.5 mm × 7.96 mm × 1.905 mm; the substrate thickness is 1.905 mm (Rogers RT/Duroid 6010 is used as the dielectric substrate material), and the maximum bandwidth is 576 MHz. In all of the previous proposed antenna designs, narrow bandwidth was achieved for Ku-band applications.

A printed double-T monopole antenna was proposed in [20]. In [21], a compact dual-band microstrip antenna for Ku-band applications was proposed, whose dimensions are 9.50 mm × 10 mm × 0.254 mm and which achieved a return loss of −23.83 dB at 12.54 GHz and −14.04 at 14.15 GHz, with a gain greater than 4 dBi. Comparatively, the proposed antenna has a limited return loss. A surface mount dual-loop antenna was proposed in [22]. A dual-band reduced-size PIFA was proposed in [23]. These antennas provided dual-band features to cover the 2.4/5.2 and 5.8 GHz WLAN bands. The limitations of these antennas were that they could not deliver a uniform omnidirectional radiation pattern. A low-cost microstrip-fed dual-frequency printed dipole antenna was proposed for wireless communications in [24]. This antenna's size is large and its bandwidth is limited. In [25], a printed dual-band dipole antenna with U-slot arms was proposed for the 2.4/5.2 GHz WLAN operation. The 370 MHz bandwidth of that antenna is insufficient to cover the desired band. In [26], a dual broadband design of a rectangular slot antenna was proposed for 2.4 and 5 GHz wireless communications. The antenna dimension is 75 mm × 75 mm, the substrate permittivity is 4.70, and the thickness is 0.80 mm. In [27], a dual-band WLAN dipole antenna using an internal matching circuit was proposed. The antenna dimensions are 12 mm × 45 mm and FR4 is used as the dielectric substrate material to cover the desired bands.

In this study, a 20 mm × 20 mm microstrip patch antenna was designed on a 1.575 mm thick Duroid 5870 substrate for use in Ku- and K-band applications. A downlink frequency of 15.56 GHz and an uplink frequency of 20.41 GHz, with return losses of −32.56 dB and −31.13 dB, respectively, a peak gain of 3.90 dB, and 98.5% average efficiency were achieved. The detailed design and simulation results for the proposed antenna were demonstrated later.

## 2. Antenna Design

**Figure 1:**
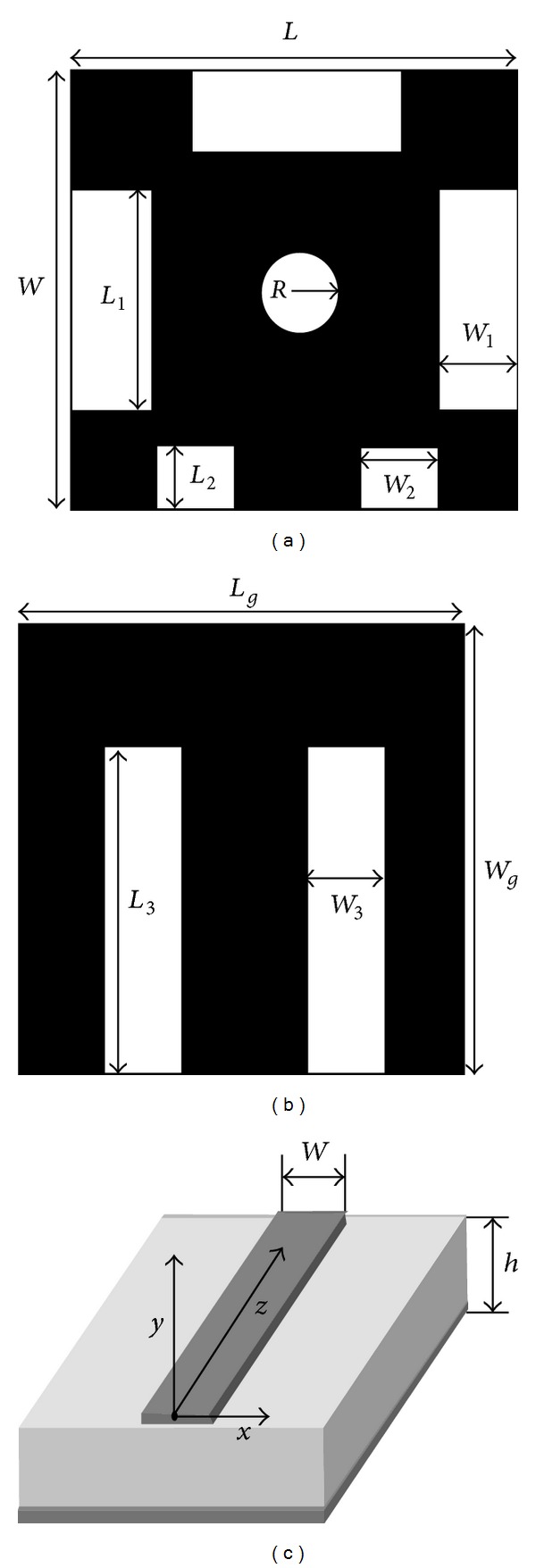
Proposed antenna: (a) top view, (b) bottom view, and (c) microstrip transmission line.

The proposed antenna design with a microstrip transmission line is shown in Figure 1. The microstrip transmission line is one type of high-grade printed circuit fabric. This line comprises a track of copper or any other conductor with an insulating substrate. The other portion of the insulating substrate contains a backbone that is made of a similar conductor. The antenna is comprised of six conducting slots on the patch and two on the ground. A circular slot and five rectangular slots are on the patch, and two rectangular slots are on the ground of the proposed antenna. The design procedure begins with the radiating patch with the substrate, the ground plane, and a feed line. The antenna is printed on a 1.575 mm thick Duroid 5870 substrate material that exhibits a relative permittivity of 2.33, a relative permeability of 1, and a dielectric loss tangent of 0.0012. A circular and five rectangular slots are cut from the rectangular copper patch. Another two rectangular slots are also cut from the ground plane. Thus, the proposed slotted circle patch antenna is finally achieved. Here, the microstrip line is used to feed the signal into the proposed antenna.

**Figure 2:**
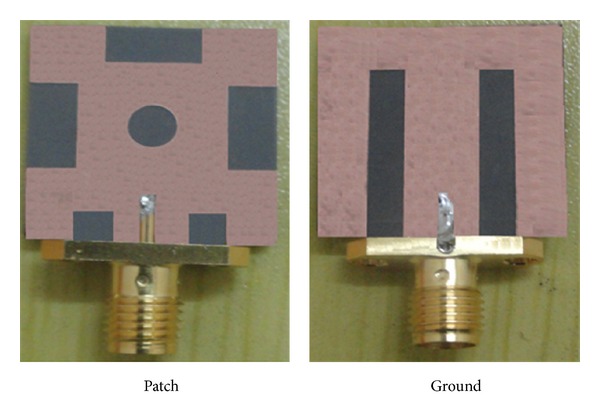
Prototype of the proposed antenna.

The subminiature version A (SMA) 50 Ω connector is used at the end of the antenna feeding line for the input RF signal. The proposed antenna prototype is shown in Figure 2. The antenna design and the dimensions were investigated by using the Ansoft HFSS software, which is based on the finite element method (FEM), and the optimal dimensions were finally determined as follows: *L* = 20 mm, *L*_*g*_ = 20 mm, *L*_1_ = 8 mm, *L*_2_ = 3 mm, *L*_3_ = 16 mm, *R* = 2 mm, *W* = 20 mm, *W*_*g*_ = 20 mm, *W*_1_ = 4 mm, *W*_2_ = 3 mm, and *W*_3_ = 3 mm.

There are different types of materials for an antenna substrate; for example, Duroid 5870 is a high-frequency laminate and PTFE (polytetrafluoroethylene) composite amplified using glass microfibers. To increase the advantages of fiber reinforcement for circuit applications and circuit producers, these microfibers have been used aimlessly. The dielectric constant of the Duroid 5870 substrate material is lower than that of other products, and it is suitable for higher-frequency bands, where dispersion and losses must be decreased because of the low dielectric loss. Due to the lack of extensive water absorption features, Duroid 5870 substrate is typically used in heavy moisture environments. The Duroid 5870 is simply cut, machined, and sheared to shape, and it is impervious to all reagents and solvents usually used in engraving printed circuit boards or plating holes and edges. Duroid 5870 has the lowest electrical loss of any amplified PTFE substrate material, lower absorption due to the fact that moisture is isotropic, and constant electrical characteristics over frequency. Duroid 5870 has been used in circuitry for commercial airline telephones, stripline and microstrip circuits, military radar systems, millimeter-wave applications, point-to-point digital radio antennas, and missile guidance systems. For these reasons, the Duroid 5870 substrate material was chosen for the proposed antenna design to achieve operation in the desired bands. The dielectric properties of the various substrate materials are listed in Table 1.

The length, width, VSWR, and return loss of the patch antenna can be calculated from (1), presented in [28], where *L* and *W* are the length and width of the patch, *c* is the velocity of light, *ε*_*r*_ is the dielectric constant of the substrate, *h* is the thickness of the substrate, *f*_0_ is the target centre frequency, *ε*_*e*_ is the effective dielectric constant, and *ρ* is the radiation coefficient:

(1) $$\begin{array}{c} W=\frac{c}{2f_{0}}\sqrt{\frac{\varepsilon_{r}+1}{2}},\\ L=\frac{c}{2f_{0}\sqrt{\varepsilon_{r}}}-2\Delta l,\\ \varepsilon_{e}=\frac{1}{2}\left(\varepsilon_{r}+1\right)+\frac{1}{2}\left(\varepsilon_{r}-1\right)\sqrt{\left(1+\frac{10h}{W}\right)},\\ \Delta l=0.412h\frac{\left(\varepsilon_{e}+0.3\right)\left[\left(W/h\right)+0.8\right]}{\left(\varepsilon_{e}-0.258\right)\left[\left(W/h\right)+0.8\right]},\\ \text{VSWR}=\frac{1+\rho }{1-\rho },\\ \text{return}\;\text{loss}=-10\log\left(\frac{1}{\rho^{2}}\right). \end{array}$$

## 3. Parametric Study

**Figure 3:**
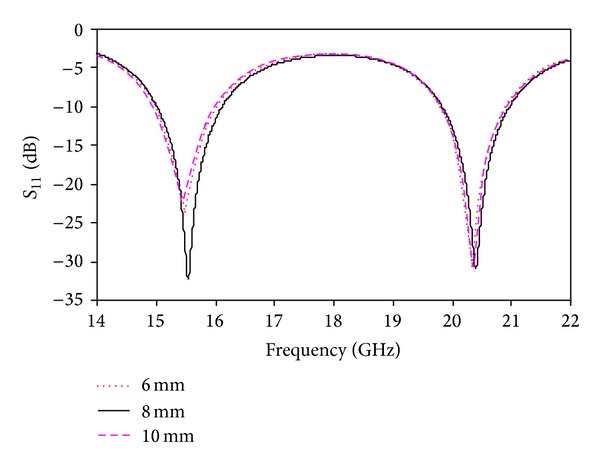
Simulated return loss for different values of *L*_1_.

**Figure 4:**
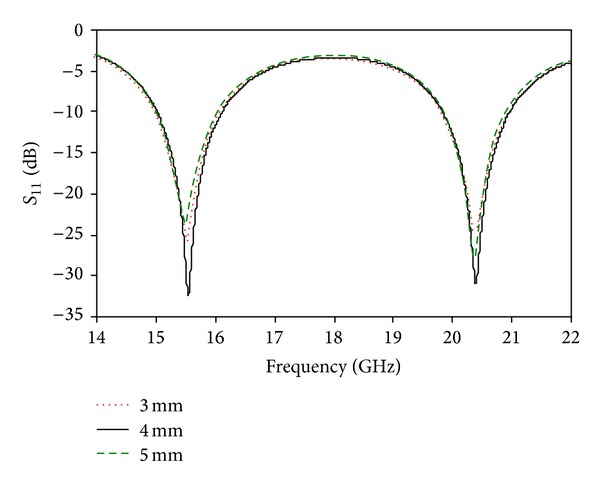
Simulated return loss for different values of *W*_1_.

**Figure 5:**
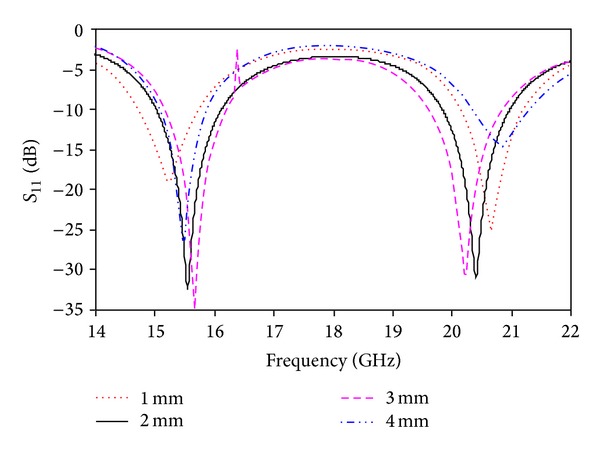
Simulated return loss for different values of *R*.

**Figure 6:**
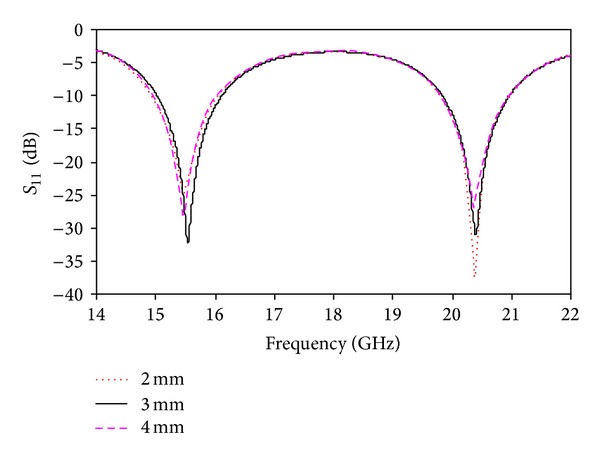
Simulated return loss for different values of *W*_2_.

**Figure 7:**
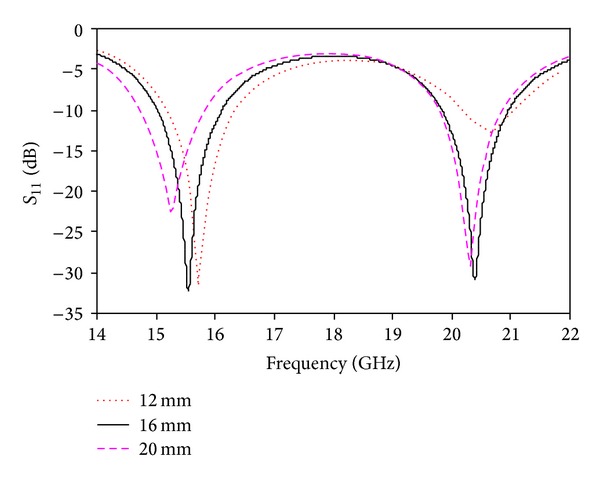
Simulated return loss for different values of *L*_3_.

**Figure 8:**
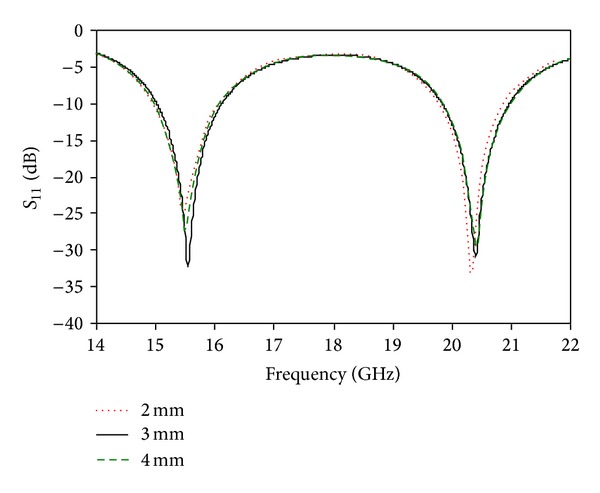
Simulated return loss for different values of *W*_3_.

A parametric study was performed to observe the effects of the proposed antenna parameters. The effects of the different parameters on the return loss were observed. In these simulations, the other parameters remained unchanged.  Figure 3 shows the simulated return loss for different values of *L*_1_. Two resonances were observed using the above values of *L*_1_, one for the lower band and another for the upper band. The return loss of the lower band is low using *L*_1_ = 6 mm and *L*_1_ = 10 mm. *L*_1_ = 8 mm can be considered the optimized value to achieve both the lower and upper bands.  Figure 4 shows the simulated return loss for different values of *W*_1_. Two resonances were found using the above values of *W*_1_. The return loss is lower for *W*_1_ = 3 mm and *W*_1_ = 5 mm than for *W*_1_ = 4 mm. *W*_1_ = 4 mm is the optimized value.  Figure 5 shows the simulated return loss for different values of *R*. The radius of the circular slot, *R*, is an important parameter that plays a role in determining the desired frequency bands. The resonances are shifted using radii with values of 1 mm, 3 mm, and 4 mm. By using *R* = 2 mm, the desired dual-band operation was obtained, with improved bandwidth.  Figure 6 shows the simulated return loss for different values of *W*_2_. By using *W*_2_ = 2 mm, the return loss is low on the lower band but high on the upper band. The return loss is low for both the upper and lower bands using *W*_2_ = 4 mm. The optimized value is *W*_2_ = 3 mm for the appropriate characteristics on both bands.  Figure 7 shows the simulated return loss for different values of *L*_3_. By using *L*_3_ = 12 mm, the return loss is high on the lower band but low on the upper band. By using *L*_3_ = 20 mm, the return loss is low on the lower band but high on the upper band. The optimized value is *L*_3_ = 16 mm. The simulated return loss for different values of *W*_3_ is shown in Figure 8. It is obvious that the optimized value of *W*_3_ is 3 mm, which is better than the other two values of 2 mm and 4 mm.

**Figure 9:**
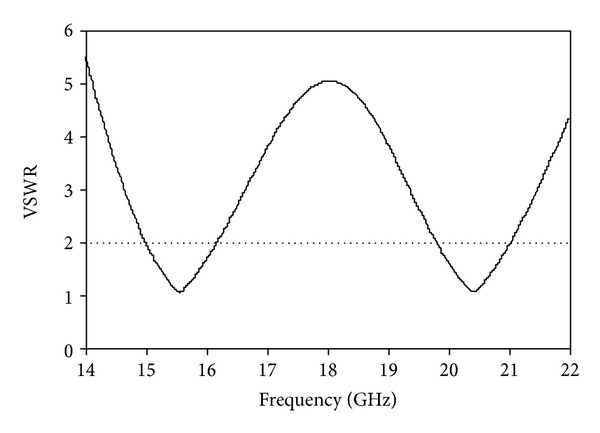
VSWR of the proposed antenna.

**Figure 10:**
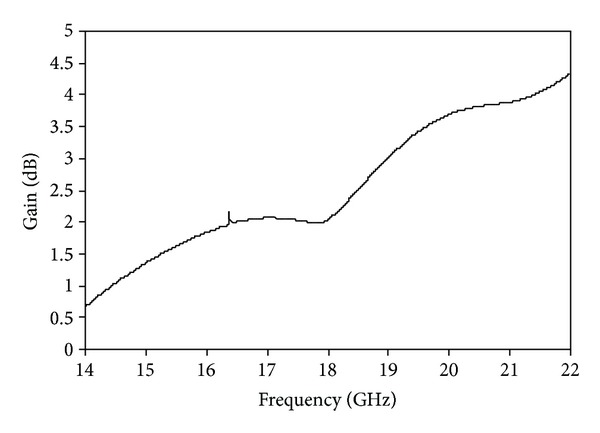
Gain of the proposed antenna.

**Figure 11:**
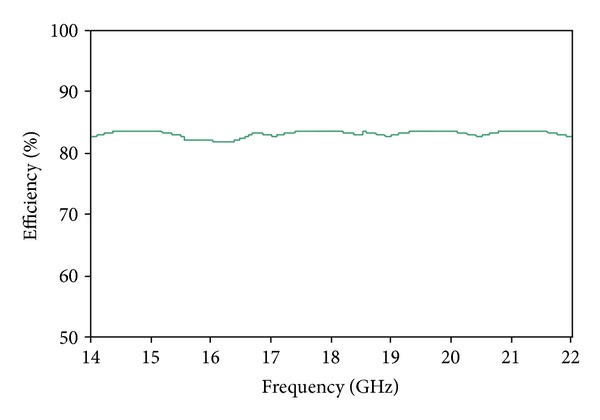
Radiation efficiency of the proposed antenna.

The simulated VSWR of the proposed antenna is shown in Figure 9. The standard value of VSWR is less than 2 for the operational bands of the microstrip patch antenna. The value of VSWR achieved is less than 2 in the desired operating bands. The simulated gain of the proposed antenna is shown in Figure 10. The achieved gains are 1.87 dB on the lower band and 3.87 dB on the upper band. The radiation efficiency of the proposed antenna is displayed in Figure 11. The 82.80% efficiency was observed over the entire operating band of Ku-and K-band applications.

## 4. Experimental Validation

**Figure 12:**
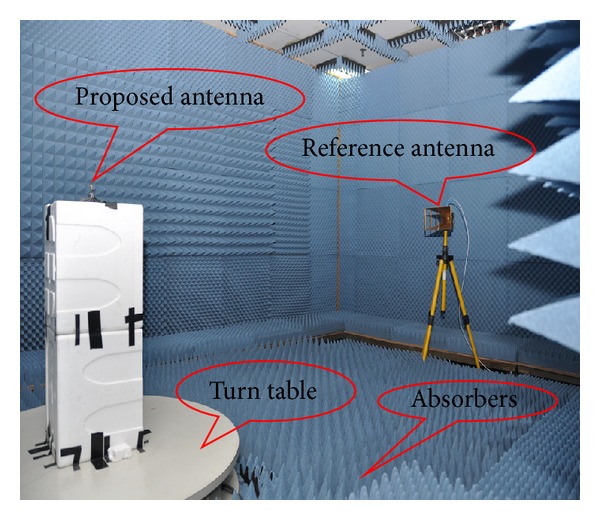
Illustration of the anechoic chamber for the proposed antenna.

**Figure 13:**
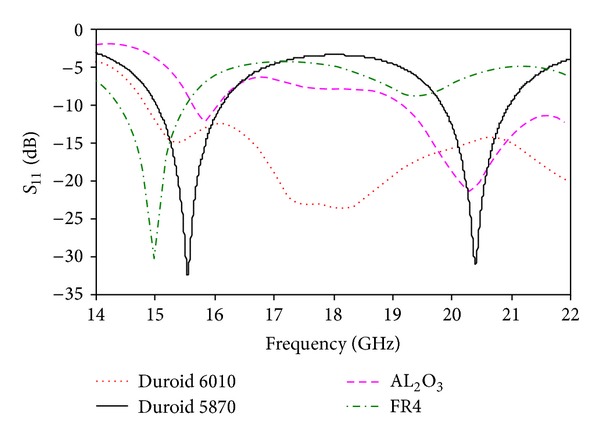
Simulated return loss with different substrate materials.

**Figure 14:**
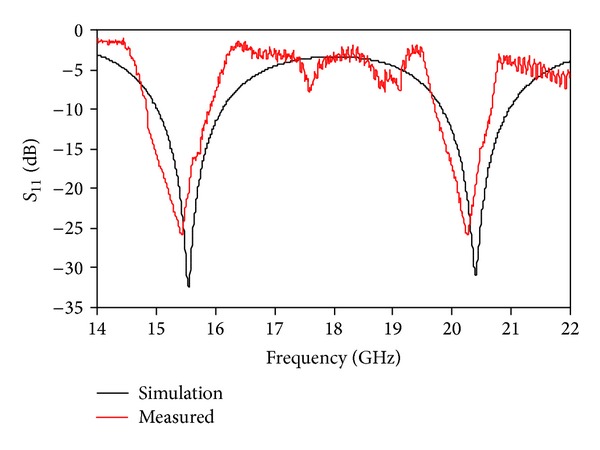
Simulated and measured return losses of the proposed antenna.

The anechoic chamber is shown in Figure 12. The proposed antenna measurement was conducted at the Microwave Laboratory, at the Institute of Space Science (ANGKASA), UKM, Malaysia. The simulated return loss with different substrate materials is shown in Figure 13. When the high permittivity material Duroid 6010 was used as a substrate, a resonance was observed but not desired. Two resonances were found using AL_2_O_3_ ceramic as the substrate material. However, the return losses of these two resonances are very low which is unwanted. When FR4 was used as a substrate, one resonance was found that is not required for dual-band operation. Finally, Duroid 5870 was used in the proposed design, and two strong resonances were achieved with both wide bandwidth and high gain. The simulated and measured return losses of the proposed antenna are shown in Figure 14. We have achieved a 1.07 GHz bandwidth, ranging from 14.86 GHz to 15.93 GHz on the 1st resonance and 0.94 GHz (19.73 GHz–20.67 GHz) on the 2nd resonance, which is validated with measurements. The resonant frequencies are shifted from 15.56 GHz to 15.46 GHz on the lower band and form 20.41 GHz to 20.27 GHz on the upper band.

**Figure 15:**
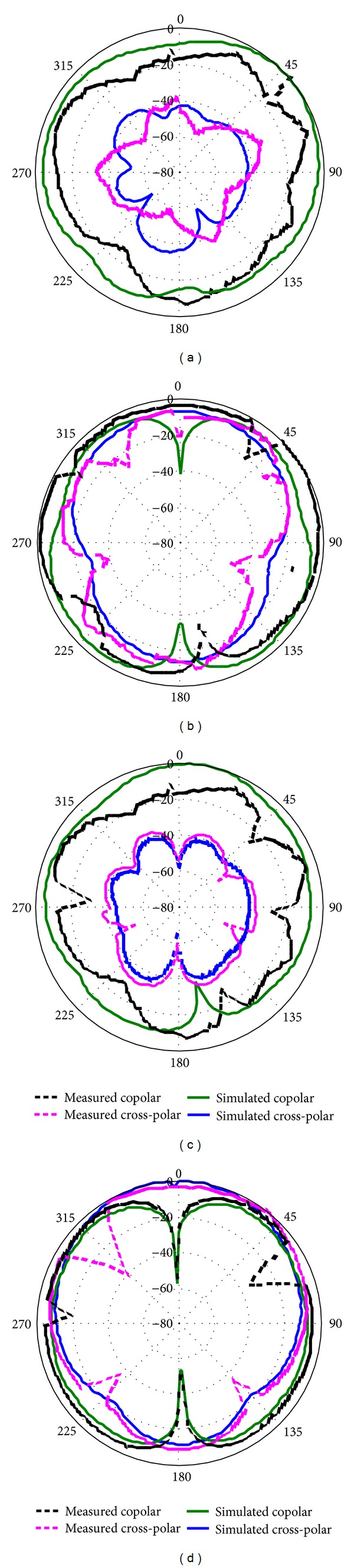
Measured radiation pattern of the proposed antenna: (a) E-plane at 15.46 GHz, (b) H-plane at 15.46 GHz, (c) E-plane at 20.27 GHz, (d) H-plane 20.27 GHz.

The measured radiation pattern of the proposed antenna is shown in Figure 15 for (a) E-plane at 15.46 GHz, (b) H-plane at 15.46 GHz, (c) E-plane at 20.27 GHz, and (d) H-plane at 20.27 GHz. Two-dimensional (2D) radiation patterns were used to specify co- and cross-polarization at 15.46 GHz and 20.27 GHz, respectively. The *E*_*φ*_ and *E*_*θ*_ fields indicate the cross-polar and copolar components, respectively. The E-plane is considered as the *y*-*z* coordinates and the H-plane as the *x*-*z* coordinates. It is desired in the case of a standard radiation pattern of a microstrip antenna that the effect of cross-polarisation is lower than that of copolarization. The cross-polarization effect is higher in the 2nd resonant frequency of 20.27 GHz. When frequency increases, the effect increases, thereby enabling the simple interpretation from the radiation pattern. From the measurement, nearly omnidirectional and symmetrical radiation patterns were attained along both the E-plane and the H-plane.

The same radiation pattern was observed to exist over the Ku- and K-bands. The obtained radiation patterns indicate that the proposed antenna delivers linear polarization, for which the level of cross-polarisation is lower than that of co-polarisation in all of the simulated radiation patterns. When the radiation pattern of a microstrip antenna is symmetric and omni-directional, it provides some reasonable benefits. One benefit is that the resonance does not shift for different directions, so a large amount of stable power is in the direction of the broadside beam. Another advantage is that the radiation pattern is more reliable on the operational bands. The level of cross-polarization at higher frequencies is comparatively higher than that at lower frequencies, which is desired due to the diffractions from the edges of the patch and ground plane. Additionally, the level of this cross-polarisation is observed to be reduced by enhancing the slots on the ground plane; in addition, the radius of the circular slot on the patch is also responsible for this effect. In this way, as will be discussed later, the enhancement of the slots is beneficial. The values of the other parameters are fixed. The results indicate that the radiation patterns are slightly shifted at the higher frequency because the distribution of the nonuniform phase is created on the proposed antenna. These radiation patterns are suitable for Ku- and K-band applications. The dimensions of the patch and the ground plane determine the radiation pattern degradation over the entire Ku- and K-bands. Thus, the sizes of the patch and the ground plane were selected carefully. If any parameter is changed, the resonant frequency is shifted. As a result, the radiation pattern is also changed from symmetric and omni-directional to bidirectional or another type. Finally, the simulation results are close to measurement results.

**Figure 16:**
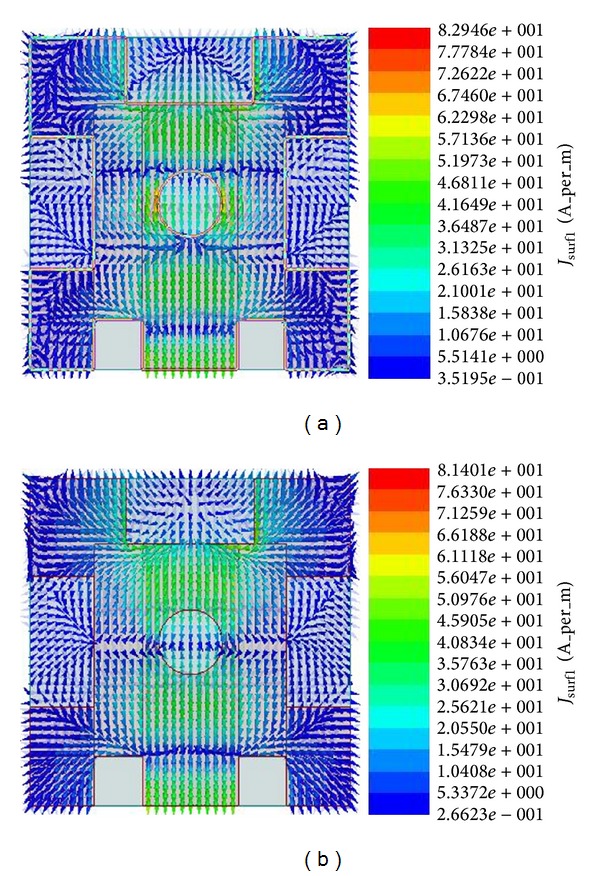
Current distribution of the proposed antenna at (a) 15.56 GHz and (b) 20.41 GHz.

Figure 16 shows the current distribution of the proposed antenna for (a) 15.56 GHz and (b) 20.41 GHz. A large amount of current flows through the feeding line. The electric field was initiated at this point. The creation of the electric field near the slots is reasonable. As a result, the excitation is strong over all the parts of the antenna for both the lower band and the upper band.

**Figure 17:**
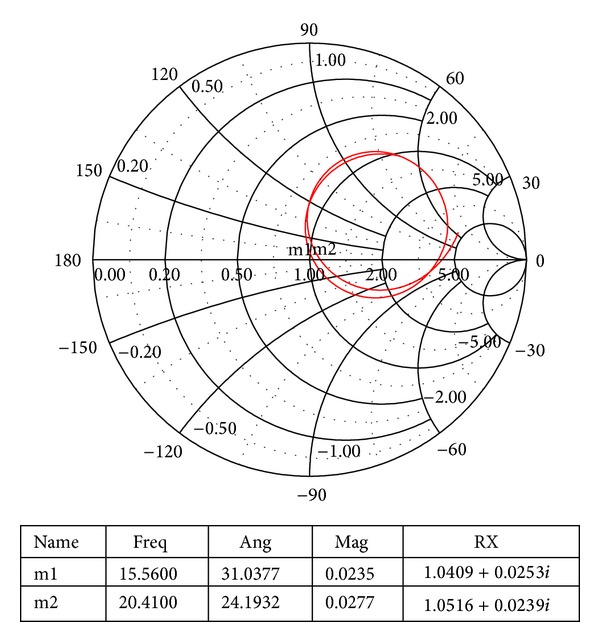
The Smith chart of the proposed antenna.

The Smith chart of the proposed antenna is shown in Figure 17. When the dimensions of the patch and the ground plane are changed, the coupling and the input impedance shift for the different resonant loops. The tightest resonant loop is found at the centre of the Smith chart of the proposed antenna design. This resonance loop indicates the improved bandwidth of the proposed antenna.

## 5. Conclusion

The proposed design and characterization of a prototype of a microstrip antenna with dual-band operation on a substrate of Duroid 5870 material to cover Ku- and K-band applications were described. The 10 dB bandwidth of 1.07 GHz in the 1st resonance at 15.46 GHz and of 0.94 GHz in the 2nd resonance at 20.27 GHz were found, which satisfies the conditions of any Ku- and K-band application. Good agreement between the simulation and measured results was observed. Attractive radiation patterns, low cross-polarization, and efficiency with improved bandwidth and higher gain make the proposed antenna compatible for use in Ku- and K-band applications.

## Figures and Tables

**Table 1:** Dielectric properties of different substrate materials.

Material	Permittivity	Loss tangent
Duroid 6010	10.2	0.0023
AL_2_O_3_	9.8	0.0009
FR4	4.66	0.020
Duroid 5870	2.33	0.0023
